# Genes associated with anhedonia: a new analysis in a large clinical trial (GENDEP)

**DOI:** 10.1038/s41398-018-0198-3

**Published:** 2018-08-13

**Authors:** Hongyan Ren, Chiara Fabbri, Rudolf Uher, Marcella Rietschel, Ole Mors, Neven Henigsberg, Joanna Hauser, Astrid Zobel, Wolfgang Maier, Mojca Z. Dernovsek, Daniel Souery, Annamaria Cattaneo, Gerome Breen, Ian W. Craig, Anne E. Farmer, Peter McGuffin, Cathryn M. Lewis, Katherine J. Aitchison

**Affiliations:** 1 Psychiatry and Medical Genetics, University of Alberta, Edmonton, AB Canada; 2 MRC Social, Genetic and Developmental Psychiatry Centre, King’s College London, London, UK; 3 Psychiatry Department, Dalhousie University, Halifax, NS Canada; 4 Division of Genetic Epidemiology in Psychiatry, Central Institute of Mental Health, Mannheim, Germany; 5 Clinical Medicine, Aarhus University, Aarhus, Denmark; 6 Croatian Institute for Brain Research, University of Zagreb, Zagreb, Croatia; 7 Psychiatry Department, University of Poznan, Poznan, Poland; 8 Psychiatry Department, University of Bonn, Bonn, Germany; 9 University Psychiatric Clinic, University of Ljubliana, Ljubljana, Slovenia; 10 Psychological Medicine, Free University of Brussels, Brussels, Belgium; 11 IRCCS, University of Brescia, Brescia, Italy

## Abstract

A key feature of major depressive disorder (MDD) is anhedonia, which is a predictor of response to antidepressant treatment. In order to shed light on its genetic underpinnings, we conducted a genome-wide association study (GWAS) followed by investigation of biological pathway enrichment using an anhedonia dimension for 759 patients with MDD in the GENDEP study. The GWAS identified 18 SNPs associated at genome-wide significance with the top one being an intronic SNP (rs9392549) in *PRPF4B* (pre-mRNA processing factor 4B) located on chromosome 6 (*P* = 2.07 × 10^*−*9^) while gene-set enrichment analysis returned one gene ontology term, axon cargo transport (GO: 0008088) with a nominally significant *P* value (1.15 × 10^*−*5^). Furthermore, our exploratory analysis yielded some interesting, albeit not statistically significant genetic correlation with Parkinson’s Disease and nucleus accumbens gray matter. In addition, polygenic risk scores (PRSs) generated from our association analysis were found to be able to predict treatment efficacy of the antidepressants in this study. In conclusion, we found some markers significantly associated with anhedonia, and some suggestive findings of related pathways and biological functions, which could be further investigated in other studies.

## Introduction

Major depressive disorder (MDD) is chronic illness which affects 350 million people world-wide according to an estimate by the World Health Organization (WHO); it is characterized by depressed mood, diminished interest, impaired cognitive function, and somatic symptoms, such as disturbed sleep or appetite. The aetiology of MDD is multifactorial with a heritability estimated to be approximately 35%^1,2^. It is generally recognized that MDD is a common illness involving multiple common genetic variants with small to moderate effect size^3^. Indeed, several large cohort-based genome-wide association studies (GWASs) in recent years have identified signals which shed new light on our understanding of MDD, for example, implicating the presynaptic protein piccolo and alpha-1 subunit of a voltage-dependent calcium channel in the pathogenesis of MDD^4,5^ and shared genetic risk for MDD, bipolar disorder and schizophrenia^6,7^. However, current studies still fall far short of accounting for all of the genetic variation in MDD with robust replicated findings. One of the possible reasons could be that the majority of these studies chose a dichotomous phenotype such as diagnosis as their outcome measure, with the currently limited understanding of the disorder leading to heterogeneity in diagnostic ascertainment^8^. Of interest, using a polygenic risk score (PRS) for schizophrenia (SCZ), Whalley et al. (2016) identified a subgroup of patients with MDD that had a higher polygenic risk of SCZ than others; this subgroup of MDD patients also showed an attenuated level of distress and neuroticism^9^. Instead of a case-control design, some studies choose quantitative traits (QTs) related to illness to increase the power of the analysis. Quantitative variables have a higher information content than categorical variables; association studies using QTs can therefore increase the statistical power four to eight-fold, with a resultant proportional reduction of the required sample size^10^. For example, one study used hippocampal atrophy measured by MRI as a QT for Alzheimer’s disease in a GWAS of only moderate sample size and nonetheless identified novel candidate loci attaining genome-wide significance^11,12^.

Different kinds of studies have long indicated that anhedonia is a fundamental feature of MDD^13,14^. DSM-IV-TR defines anhedonia as diminished interest or pleasure in response to stimuli that were previously perceived as rewarding during a premorbid state^15^. Moreover, anhedonia has been shown to be able to predict a longer time to remission and fewer depression-free days^16,17^. Specifically, using the same dataset as in our present study, Uher et al. showed that out of the six disease dimensions (mood, anxiety, pessimism, interest-activity, sleep, and appetite), the interest-activity dimension (anhedonia) at baseline was the only dimension able to predict poor treatment outcome in the later time points^17^. Both twin and family studies demonstrate that 44% of anhedonia is attributable to genetic factors, especially additive genetic effects, and first-degree relatives of patients with MDD display anhedonia-related phenotypes when compared to controls^18,19^. Although different threads of evidence have validated anhedonia as a QT of MDD, no genetic or genomic study has yet been carried out to identify candidate loci associated with this key feature of MDD.

Our study used a dimensional score of anhedonia to conduct a GWAS and to estimate the heritability of this phenotype accounted for by common variants, aiming to shed new light on our understanding of MDD.

## Materials and methods

### Patient recruitment

Seven hundred and ninety-six people (296 males, 500 females) with unipolar depression of at least moderate severity according to ICD-10 (International Classification of Diseases, 10th revision, Mental and Behavioural Disorders, Research Criteria) and DSM-IV (Diagnostic and Statistical Manual of Mental Disorders, fourth edition) criteria^20^ were recruited from eight European countries in the GENDEP project^21^. All patients were of European ancestry without a family history of schizophrenia, bipolar disorder or a current dependency on alcohol or drugs. For further details about the GENDEP project, see Uher et al.^21,22^.

### Phenotype definition

Uher et al. conducted factor analysis of depression severity data generated from three measures: the Montgomery-Asberg Depression Scale (MADRS); the Hamilton Depression Rating Scale (HDRS) and the Beck Depression Inventory (BDI). Although these measures had previously been widely used in studies of depression, prior to GENDEP, no study had used all three measures simultaneously. Six dimensions with continuous factor scores representing the different aspects of the psychopathology of depression were extracted from initial questionnaire estimates^23^. Of these six dimensions, the interest-activity score at baseline (which had higher information loadings from items in the three measures relevant to anhedonia, such as “inability to feel”, “lassitude” in the MADRS; “sexual interest” in the HAMD-17; “enjoyment” and “interest in people” in the BDI) was found to significantly predict response to treatment with the antidepressants used in the study^17^. In this analysis, we used the baseline interest-activity score as our outcome measure for GWAS.

### DNA extraction and genotyping

DNA was extracted from blood samples collected in ethylenediaminetetraacetic acid (EDTA) tubes using standard procedures^24^; genotyping was performed in the Centre National de Génotypage using the Illumina Human610-quad bead chip (Illumina) as described^25^.

### Genotype quality control and population stratification analysis

Standard steps were taken for quality control of genomic data in PLINK 1.09^26^ and data were excluded on failure to pass the following thresholds: consistency of gender information between genomic data and demographic data, SNP genotyping rate ≥ 95%, individual genotyping rate ≥ 97%, Hardy–Weinberg equilibrium test (*P* ≥ 0.001), minor allele frequency (MAF) ≥ 0.01. Furthermore, using both PLINK 1.09 and KING^27^, pairwise identity-by-state (IBS) was calculated and outliers or subjects showing unknown familial relationship with others (proportion IBD *>* 0.05) were subsequently excluded^26,28^.

### Population stratification analysis

Population stratification analysis was conducted using EIGENSTRAT^29^, which employs principal component analysis (PCA) to capture hidden population structure in genomic data. Prior to the analysis, data were pruned to make sure adjacent SNPs were in no more than weak linkage disequilibrium (LD) with each other (PLINK command: --indep-pairwise 50 10 0.5)^30^. This generated 20 principal components (PCs) which were controlled for as covariates in the subsequent association analysis.

### Imputation of missing genotypes using the 1000 Genomes dataset

Following quality control steps, imputation was carried out on genomic data. We employed IMPUTE2 + SHAPEIT2 to impute using the 1000 Genomes phase 3 dataset as the reference dataset^31,32^. Before imputation, the physical position of SNPs was updated using UCSC Liftover tool (https://genome.ucsc.edu/)^33^ to the haploid human genome build 19 (hg19). Following imputation, the same quality control steps were used to clean the resultant imputed data.

### GWAS using a linear mixed model (LMM)

In order to test for genotype–phenotype association while controlling for potential confounding factors such as population structure, family structure, and cryptic relatedness simultaneously, we used factored spectrally transformed LMM (FaST-LMM) for our association study^34^. In brief, the LMM log likelihood of the phenotype data, *y* (dimension *n* × 1; *n* denoting the cohort size), given fixed effects *X* (dimension *n* × *d*; *d* denoting the number of fixed effects in a single model, including the offset, the covariates, and the SNP to be tested), can be written as

1 $$LL\left( {\delta _e^2,\,\delta _g^2,\,{\boldsymbol{\beta }}} \right) = \,{\mathrm{logN}}\,\left( {{\boldsymbol{y}}\left| {\boldsymbol{X}} \right.\beta ;\,\delta _g^2{\boldsymbol{K}}\, + \,\delta _e^2{\boldsymbol{I}}} \right)$$

where *N* (***r|m***; Σ) denotes a normal distribution of variable ***r*** with mean ***m*** and covariance matrix Σ; ***K*** (dimension *n* × *n*) is the genetic similarity matrix; ***I*** is the identity matrix; $\delta _e^2$is the magnitude of the residual variance; $\delta _g^2$is the magnitude of the genetic variance; and ***β*** (dimension ***d*** × 1) denotes the weight of the fixed effects.

The “Fa” in FaST-LMM stands for factorization. Let S be genetic similarity matrix, as the covariance matrix of the normal distribution becomes a diagonal matrix ***S*** + *δ****I*** (spectral decomposition), the log likelihood can be rewritten as the sum over *n* terms. Factorization dramatically increases the size of datasets that can be analyzed with LMM, and additionally enhances the speed and feasibility of the analysis.

In our analysis, we chose the continuous interest-activity score as our outcome measure, controlling for gender, age, years of education, recruitment centres and the first 20 PCs from EIGENSTRAT as covariates.

### Replication analysis using STAR*D

Following our initial findings, we used data from the Sequenced Treatment Alternatives to Relieve Depression Study (STAR*D) to replicate our primary results. Detailed information about the STAR*D including its demographic characteristics and genomic profile have been previously described^35–37^. In brief, 1351 patients with MDD were recruited with the phenotype being defined as the sum of items with corresponding content in baseline HAMD-17, QIDS-SR, QIDS-C and the research outcome assessor-rated 30-item Inventory for Depression Symptomatology^17^, with genomic profile including 7405247 SNPs after quality control and imputation^38^. Further, a linear model using PLINK 1.09 was chosen with age, gender, years of education, recruitment centre, and the first four population PCs being included as covariates.

### Gene-based and pathway analysis

Emerging evidence has suggested that disease- or trait-associated genetic variants identified from GWASs tend to be enriched in genic regions including multiple associated variants at a single locus^39,40^. Therefore, we utilized fastBAT which stands for a fast and flexible set-Based Association Test and the *P* values from the LMM analysis for gene-set testing^41^ to discover genes associated with the interest-activity score based on the aggregated effect of a set of SNPs (e.g., SNPs within or close to a gene) with their generated *P* values being adjusted using Bonferroni correction (0.05/22484).

### Biological interpretation, heritability, and genetic correlation estimates

In order to further understand the resultant signals and their associations with the interest-activity score, we chose loci with an association *P* value less than 1 × 10^*−*5^ and used DEPICT (Data-driven Expression Prioritized Integration for Complex Traits) to accomplish gene prioritization and tissue/cell type enrichment analysis with a false discovery rate (FDR) set as 1%^42^. Recent studies have shown that mutation-intolerant genes which are presumed to hold critical biological functions are enriched in rare variants in psychiatric disorders such as autism and intellectual disability (ID)^43,44^; this pattern also extends to both rare and common variants for schizophrenia^45^. To test whether it also holds for common variants in our MDD-related phenotype, we investigated the enrichment of genes harboring SNPs attaining an association *P* value ≤ 10^*−*5^ in the set of loss-of-function (LOF) genes characterized by the Exome Aggregation Consortium (ExAC), setting the constraint metric pLI ≥ 0.9 (probability of being LoF intolerant) according to their recommendation^46^.

Furthermore, aiming to detect phenotypic variance explained by common SNPs (*h*_*g*_) in our sample and to explore traits which shared a common genetic effect with the interest-activity score, we chose LDSc (LD score regression)^47^ from LD Hub—a centralized database of summary-level GWAS results for 177 diseases/traits from different publicly available resources/consortia and a web interface that automates the LD score regression analysis pipeline for detection of *h*_*g*_ and genetic correlation between target phenotype and multiple traits^48^.

### Association analysis with longitudinal change of anhedonia following treatment with antidepressants

The baseline interest-activity score from the study by Uher el al.^17^, chosen as the primary outcome measure in our GWAS, was found to significantly predict treatment response in both GENDEP and STAR*D. In order to investigate the potential association between the SNPs associated with the baseline interest-activity score and longitudinal change in the score, we summed up all the associated SNPs to calculate a unweighted PRS for each individual, then conducted association analysis between this PRS and the interest-activity score from week 1 to week 10 using a LMM. The fixed effects of the model included our predictor (PRS) and covariates (age, quadratic effect of age, gender, baseline interest-activity score, and centerid) while the random effect included a random intercept and a random time effect (slope). The PRS was generated using PLINK^26^ and the above-mentioned association analysis was implemented using the package “nlme” in an R environment^49^.

## Results

### Demographic characteristics and genome-wide association analysis

#### Demographic characteristics

Of 796 people with genomic data, 759 had a baseline interest-activity score derived from factor analysis (286 males, 473 females). The mean age was 42.05 (11.59), mean years of education 12.31 (3.12), mean baseline MADRS 28.90 (6.77), mean baseline HDRS 21.88 (5.24), and mean baseline BDI was 28.10 (9.76).

#### Genome-wide association analysis

**Fig. 1:**
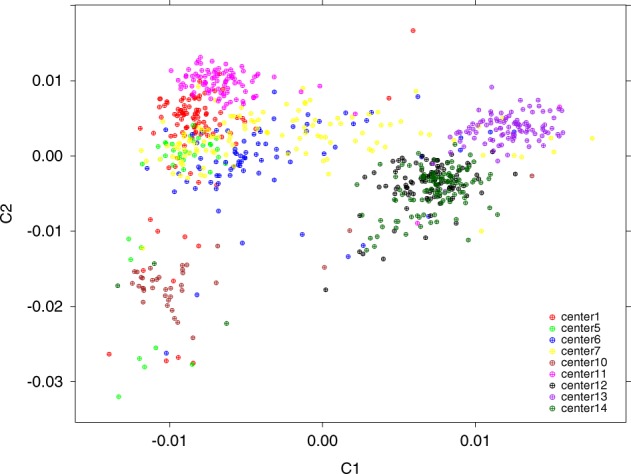
Population scatter plot of PC1 and PC2 stratified by centre

After imputation and quality control, 1,313,135 SNPs (of which 789,990 were imputed with high-quality imputation, i.e., info > 0.6, LD pruning at *R*^2^ < 0.5) in 760 individuals remained in the present analysis and as shown in Fig. 1, all study subjects were of European ancestry with no gross population stratification.

**Fig. 2:**
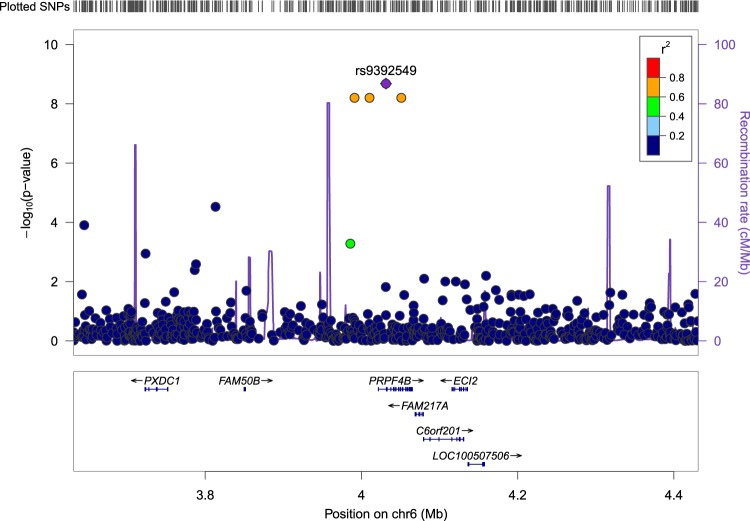
Regional association plot of the SNP, rs9392549, that attained the lowest *P* value (2.07 × 10^*−*9^) in the association analysis

**Fig. 3:**
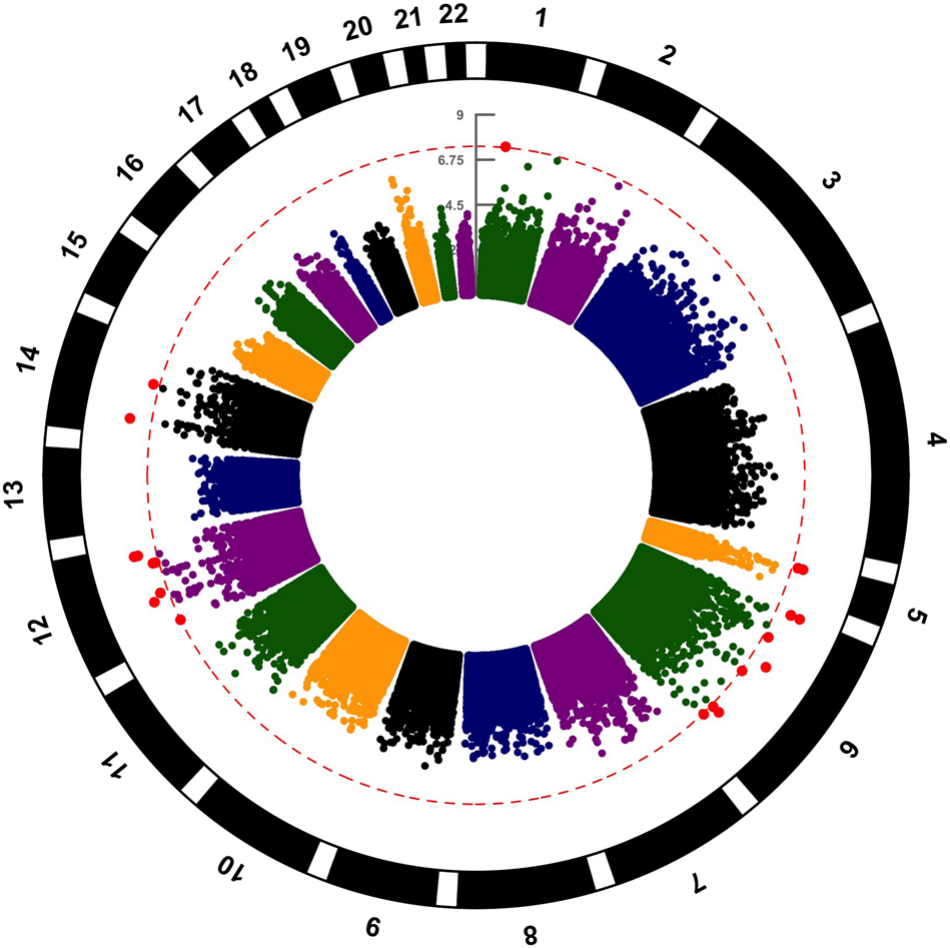
Manhattan plot for the post-imputation GWAS on interest-activity score

**Fig. 4:**
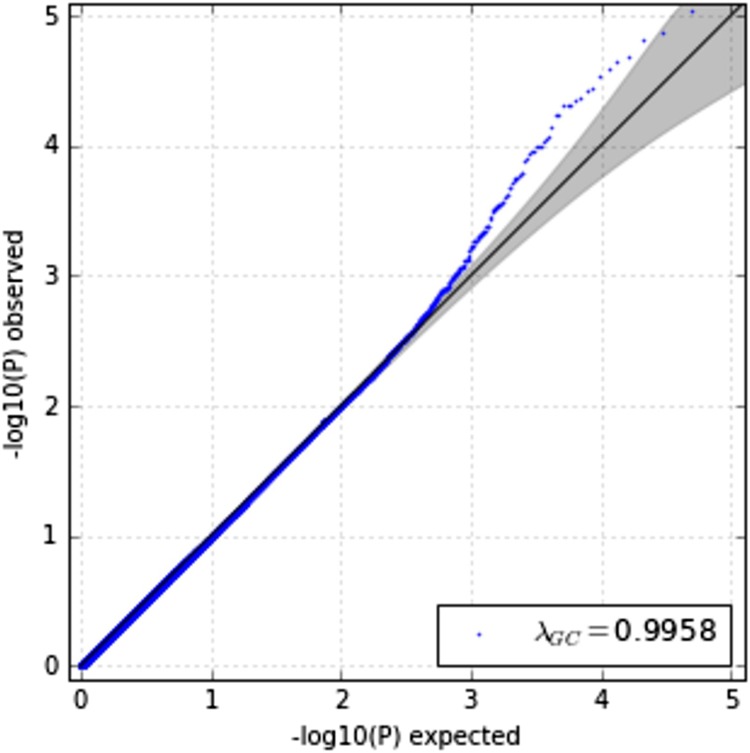
Quantile-Quantile (QQ) plot for the post-imputation GWAS of the interest-activity score

Association analysis of interest-activity scores using LMM identified 18 SNPs that passed genome-wide significance (5 × 10^*−*8^) when including gender, age, years of education and 20 PCs of the population structure from EIGENSTRAT as covariates. The top SNP from the analysis, rs9392549, in an intronic region of *PRPF4B* (pre-mRNA processing factor 4B) located on chromosome 6, had a *P* value of 2.07 × 10^*−*9^ (Figure 2). Table 1 summarizes the top signals from the association analysis and Fig. 3 displays this as a circularized Manhattan plot. The genomic inflation factor (*λ*) was calculated as an index of any potential confounding effect in the analysis, and the results were consistent with potential confounding effects having been adequately covered (*λ* *=* 0.9958, Fig. 4).

**Table 1:** Significant signals arising from the GWAS of the interest-activity score in GENDEP and corresponding replication in STAR*D

SNP	Chr	ChrPos	MAF	Reference allele	Position	Nearest gene	Impute info	GENDEP (discovery stage)			STAR*D (replication stage)		
								P Value	Beta	SE	P value	Beta	SE
rs9392549	6	4031372	0.01974	A	Intronic	PRPF4B	0.987	2.07E−09	0.19	0.03	0.031	0.22	0.1006
rs650466	12	118646661	0.01581	T	Intronic	TAOK3	0.905	2.83E−09	0.19	0.03	0.576	0.0715	0.1278
rs10498321	14	34194231	0.1303	C	Intronic	NPAS3	1	3.08E−09	0.19	0.03	0.8461	−0.01086	0.05596
rs116367381	6	65987638	0.01008	T	Intronic	EYS	0.641	4.42E−09	0.19	0.03	0.5175	−0.1005	0.1552
rs146142861	12	50821551	0.01546	T	Intronic	LARP4	0.642	5.46E−09	0.19	0.03	NA	NA	NA
rs77266588	6	142944380	0.02905	C	intronic	LOC153910	0.9	1.16E−08	0.18	0.03	0.05122	−0.1913	0.1831
rs147128318	12	61824491	0.01273	T	intergenic	FAM19A2	0.669	1.52E−08	0.18	0.03	NA	NA	NA
rs112593182	14	71425668	0.01184	T	intergenic	COX16	0.827	1.85E−08	0.18	0.03	0.2964	−0.1913	0.1831
rs7732588	5	19767062	0.01598	A	intronic	CDH18	0.9	1.90E–09	0.18	0.03	0.3448	−0.1127	0.1193
rs831431	12	104029996	0.02237	C	intronic	STAB2	0.961	1.92E−08	0.18	0.03	0.046^a^	−0.32	0.1598
rs80074882	6	32184329	0.02078	G	intronc	NOTCH4	0.69	2.09E−08	0.18	0.03	0.8249	0.03	0.1225
rs73187882	12	104013517	0.02656	G	intronic	STAB2	0.9	2.57E−08	0.18	0.03	0.9875	0.001458	0.09282
rs11755163	6	142680533	0.06824	G	intronic	ADGRG6	0.663	2.88E−08	0.18	0.03	0.3793	0.07402	0.08417
rs117555972	6	82161785	0.01123	C	intergenic	FAM46A	0.796	3.30E–08	0.18	0.03	0.6524	0.05853	0.1299
rs78164217	6	155798386	0.01189	G	intergenic	COX16	0.765	3.33E−08	0.18	0.03	0.9439	0.009437	0.1341
12:15021349	12	15021349	0.01126	A	NA	NA	0.751	3.79E−08	0.18	0.03	NA	NA	NA
rs118166313	6	155772619	0.01583	T	intronic	NOX3	0.771	4.18E−08	0.17	0.03	0.9499	0.008294	0.1319
rs138600970	6	23054276	0.01061	A	intronc	LOC105374974	0.651	4.98E−08	0.17	0.03	0.1227	0.2776	0.1798

^a^rs118190482 (in LD with rs831431, *R*^2^ = 0.5)

The replication analysis using the STAR*D dataset indicated that while none of the associated SNPs found in the GENDEP dataset were replicated at a Bonferroni-adjusted significance level (0.03/18 = 0.0016); two of them, the top signal (rs9392549) and rs118190482 located in the intronic region of *STAB2* (in LD with rs831431, *R*^2^ = 0.5), were nominally significant (*P* = 0.03 and 0.046 respectively, in Table 1).

### Gene-based and gene prioritization analysis

Gene-based association analysis indicated no gene was associated at gene-level significance (*P* value = 2 × 10^*−*6^). The gene with the strongest signal from the analysis was *KITLG* on chromosome 12 (KIT ligand, *P* value = 3.09 × 10^*−*5^).

Using DEPICT, one SNP, rs1001415, which is intronic in *EFCAB2* (EF-hand calcium binding domain 2) on chromosome 1, was prioritized owing to sharing more similar biological functions with other associated loci, although the *P* value was only at a trend level (nominal P = 0.09). Interestingly, Westra et al. reported that rs1001415 is in high LD with a cis eQTL SNP (rs4658697) in an intronic region of a transcript (NM 001143943.1) of *EFCAB2*^50^. Furthermore, gene-set analysis found one gene ontology item (GO:0008088), axon cargo transport, was over-represented by associated loci from our association analysis with a nominal *P* value being 1.15 × 10^*−*5^. Cell/tissue annotation analysis saw our associated loci were highly annotated in the MeSH first term of “hypothalamus” and the MeSH second term of “nervous system” (nominal *P* = 0.004). Although some results generated from DEPICT showed nominal significance, they failed to reach FDR. Nevertheless, our target genes were shown to be significantly enriched by the gene set (3203 genes) characterized by ExAC as mutation intolerant (*P* = 0.001).

### Heritability estimation and genetic correlation analysis

Estimation of *h*_*g*_ showed that 69% of the phenotypic variance of the interest-activity dimension in our sample could be explained by common SNPs (*h*_*g*_ *=* 0.69 *±* 0.88). As shown in Table S1 and Figure S1, the genetics of the interest-activity score was highly positively correlated with Parkinson’s disease (PD) (rg = 0.83, se = 1.14), and with Alzheimer’s disease (rg = 0.43, se = 0.32). Moreover, its genetics was negatively correlated with that of the gray matter volume of nucleus accumbens (rg = −0.6492, se = 0.84), eczema (rg = −0.41, se = 0.44) and with subjective well-being (rg = −0.32, se = 0.47). This is consistent with a pleiotropic effect. However, the results should be interpreted with caution given that none of the *P* values generated from our genetic correlation analyses reached the statistical significance of 0.05.

### Association analysis between the PRS and longitudinal change of anhedonia up to ten weeks following antidepressant treatment

The association analysis showed that the PRS calculated based on the GWAS of baseline interest-activity score was significantly associated with longitudinal change of anhedonia following antidepressant treatment (*β* *=* 1.73, *P* = 0.0023). In order to evaluate if the top hit (rs9392549) from the GWAS of baseline interest-activity score solely drove the identified association, we conducted a secondary analysis using same model conditioning on rs9392549; the association between the PRS and longitudinal change of anhedonia remained significant (*β* *=* 1.64, *P* = 0.0091).

## Discussion

To the best of our knowledge, this is the first genome-wide association analysis of anhedonia in patients with MDD. We used a LMM to conduct the association analysis, which identified 18 SNPs of genome-wide significance, with the most significant being rs9392549 in an intronic region of *PRPF4B* on chromosome 6 (*P* = 2.07 × 10^*−*9^). Although no gene was significant on gene-set testing, gene prioritization analysis found one intronic SNP (rs1001415) in *EFCAB2* to be significant with a trend (*P* = 0.09) and the associated loci showed enrichment for a particular gene ontology locus, axon cargo transport (GO:0008088). Furthermore, using LD regression, we showed that 69% of the variance in our phenotype was explained by common SNPs and the markers associated with anhedonia were positively correlated with PD and with Alzheimer’s disease, while being negatively correlated with nucleus accumbens gray matter volume.

The use of a LMM for the genome-wide association analysis is in contrast to the classic general linear model (GLM) in how population stratification or other sample structure issues are addressed. Such confounding factors are detected and addressed in GLM by using genomic control^51^, ancestry inference (analysis of populationstructure)^52–54^ and PCA^29,55^. However, these strategies fail to account for sample features such as family structure or cryptic relatedness; for population stratification owing to ancient population divergence, methods like genomic control are relatively weak^56^. Linear mixed modeling by contrast fits population structure as a fixed effect and a similarity matrix between individuals as the variance-covariance structure of the random effect^57^; such a method has been shown to yield more a conservative *λ*_GC_ compared to other approaches^57,58^. Using a similar statistical model, the CONVERGE consortium conducted a genome-wide association analysis in a large cohort of Chinese female patients with severe MDD, with two significant loci being identified and replicated in different samples^59^. These two loci (rs12415800 and rs35936514 on chromosome 10), however, were not replicated in our study given the rarer frequency of these loci in the European population.

One intronic SNP (rs9392549) in *PRPF4B* yielded the lowest *P* value in association with anhedonia (*P* = 2.07 × 10^*−*9^, replicated *P* = 0.03). *PRPF4B*, pre-mRNA processing factor 4 homolog B, is a kinase involved in mRNA splicing that is involved in biological pathways such as inositol phosphate metabolism^60^. Patients with MDD have been shown to have alterations in mRNA splicing, especially in that of neurotransmitter receptors^61,62^. For instance, in suicide victims with a history of major depression, adenosine-to-inosine RNA editing within the coding sequence of the serotonin 2C receptor (5-HT_2*C*_) pre-mRNA was significantly decreased and this effect was reversed by treatment with the antidepressant fluoxetine^63^. Additionally, inositol phosphate has been repeatedly implicated in the pathophysiology of affective disorders including MDD, with potential new treatments arising^64–66^. For example, a double-blind, controlled clinical trial in MDD indicated that the overall improvement in scores on the Hamilton Depression Rating Scale was significantly greater for inositol than for placebo after 4 weeks of treatment^67^.

One of two associated loci which were replicated with a nominal significance, rs831431 (*P* = 1.92 × 10^*−*8^, replicated *P* = 0.046) is a brain eQTL located in the intronic region of *STAB2*, which encodes stabilin 2. Stabilin 2 plays a critical role in angiogenesis^68^. According to BRAINEAC^69^, rs831431 significantly affects the expression of one of *STAB2*’s transcripts (tID = 3429159), especially in the thalamus (eQTL *P* = 0.01). Although the precise role of *STAB2* in the pathogenesis of MDD or anhedonia still remains unclear, it could be hypothesized that deficits in neuroplasticity, potentially mediated by abnormal angiogenesis lead to dysfunction in pleasure-rewarding circuitry. This could be in a temporal-specific manner, analogous to the time-dependent gene expression that is commonly seen in genes related to neurodevelopment^70^.

Of the other associated loci, rs10498321 is in an intronic region of *NPAS3*. *NPAS3*, neuronal PAS domain protein 3, is a brain-enriched transcription factor, expression deficits in which can cause deficiency in neurogenesis, especially in the hippocampus^71^.

To date, *NPAS3* has been mainly studied in schizophrenia and bipolar disorder^72–74^ and schizophrenia, especially with negative symptomatology, is another condition in which anhedonia may be a common feature; to our knowledge, this is the first report of an association between *NPAS3* and a MDD-related phenotype. Intriguingly, one of the top signals (rs7973260)^75^ identified in a GWAS of depressive symptoms in a large cohort from the UK Biobank is in the 18 kb downstream of rs650466, quasi-replicating the current finding and highlighting the potential importance of this genomic region in understanding the biological mechanism of MDD.

Given the modest replication using STAR*D, we carried out a genetic correlation analysis between STAR*D and GENDEP by executing the “sumsum” command in PRSice^76^, which takes respective summary statistics as input. The result displayed in Figure S2 indicated that although the two datasets were significantly correlated with each other at multiple *P*-value thresholds (*P*_*T*_ at 0.04, 0.05, 0.2, 0.3, and 0.5), the variance explained by each other (*R*^2^) was relatively small, which may at least partly explain the relatively weak replication signal in STAR*D. Although it has been widely thought that QTs underpinning the symptomatology of psychiatric disorders could increase the power of the identification of risk variants, the way in which QTs are established has been inconsistent. Of note, the QT of anhedonia was defined in contrasting ways in GENDEP and STAR*D owing to differential measures available. While our study provides an alternative approach for GWAS with limited sample size, it points to the importance of future efforts to validate different measures of QTs along the lines of the RDoC strategy^77^.

In our gene prioritization analysis, one intronic SNP (rs1001415) in *EFCAB2* was found to be more similarly associated with other associated loci in terms of biological function. *EFCAB2*, EF-hand calcium binding domain 2, is located in SOR (smallest overlapping region) at 1q44 with three other genes: *HNRNPU*, *FAM36A*, *NCRNA00201*. Patients with microdeletions of this region display ID and seizures^78,79^, which implies a role in neurodevelopment and cognitive function. Of note, it is in high LD with one cis eQTL SNP (rs4658697); therefore, we suggest that future studies could use rs1001415 as a proxy for rs4658697 for the expression of *EFCAB2*. In addition, one gene set (GO:0008088, axon cargo transport) was over-represented by our associated markers. It is therefore possible that dysfunctional axon cargo transport affected by our identified genes in brain regions relevant for reward circuitry^80,81^ may be associated with impaired neurotransmitter release (dopamine, etc.), putatively leading to anhedonic symptoms.

Although the cross-phenotype LD score regression failed to generate a genetic correlation with a significant *P* value, it provided a trend worth further elaboration. Specifically, anhedonia in our study was positively correlated with PD (rg = 0.8). In fact, anhedonia independent of clinical diagnosis and PD are both dopamine-dependent processes and anhedonia is one of the most commonly observed non-motor symptoms in PD^82,83^. Moreover, anhedonia was negatively correlated with nucleus accumbens gray matter volume (rg = −0.6). The accumbens is a key structure in the reward circuit; structural and functional changes in the accumbens have been repeatedly implicated in substance abuse-related and MDD-related anhedonia^84,85^. Nonetheless, any inference from our current findings should be made with the caveat that due to the lack of statistical significance, potential type I error (false positive error) cannot be excluded.

Furthermore, the significant association detected between our PRS and the longitudinal change in anhedonia is of interest in that it appears to offer insight not only into the polygenic underpinnings of anhedonia but also into its change during treatment. This preliminary association analysis of the PRS generated by our association findings illustrates the potential of applying such a polygenic profile to better our prediction of treatment response. This could be further tested in the response to treatment of other disorders in which anhedonia is also a feature.

## Strengths and limitations

Strengths of our study include the LMM which controls for confounding factors such as population stratification and cryptic relatedness in a perhaps more robust manner than GLM. However, there are limitations. Firstly, the sample size for our study is modest. Generally, the majority of power calculations used for GWAS employ a case-control design; the use of an endophenotype such as anhedonia, a QT of complex disease biologically hypothesized to be closer to underlying genetic variation, should increase the power of association^10^. Many approaches for linear mixed modeling of GWAS are computationally challenging, which makes such methodology less popular for GWAS of large sample sizes. Our study provided another new association strategy for GWAS of modest sample sizes, although replication of significant signals in a larger independent sample is required.

Secondly, 16 out 18 SNPs identified in the association analysis have a MAF lower than 0.03. The MAF distribution of our genomic data indicated that 67% of alleles fall into the interval between 0.01 and 0.05 (Figure S3). Enrichment of signals in the lower bound of the MAF spectrum is methodologically recognized; we are aware that given the sample size, these associations may be false positives (a “winner’s curse”), as the number of individuals with a minor allele is very limited.

Thirdly, not all patients were drug-free at the time of recruitment (baseline), some medications such as antidepressants^86,87,88^ or benzodiazepines^89^ etc. might affect patients’ anhedonia level at the baseline.

## Conclusion

In summary, this first GWAS of anhedonia in MDD identified a number of SNPs attaining genome-wide significance. The top hits include loci such as *NAPS3* which has been associated with schizophrenia, another condition in which anhedonia may be a prominent feature. It is therefore possible that our findings are relevant not only for anhedonia in MDD, but also for anhedonia in other neuropsychiatric conditions. Consistent with this, cross-phenotype correlation analysis gave suggestive signals for PD and nucleus accumbens size. We suggest that further genetic exploration of anhedonia in MDD and other disorders could be a new and productive avenue that could lead to new treatments for this disabling feature of many neuropsychiatric conditions.

## Electronic supplementary material

Figure S1

Figure S2

Figure S3
