# Supplementary figures and images for: Genes associated with anhedonia: a new analysis in a large clinical trial (GENDEP)
Source: Transl Psychiatry. 2018 Aug 13;8:150. doi: 10.1038/s41398-018-0198-3 (PMC6089928; doi:10.1038/s41398-018-0198-3)

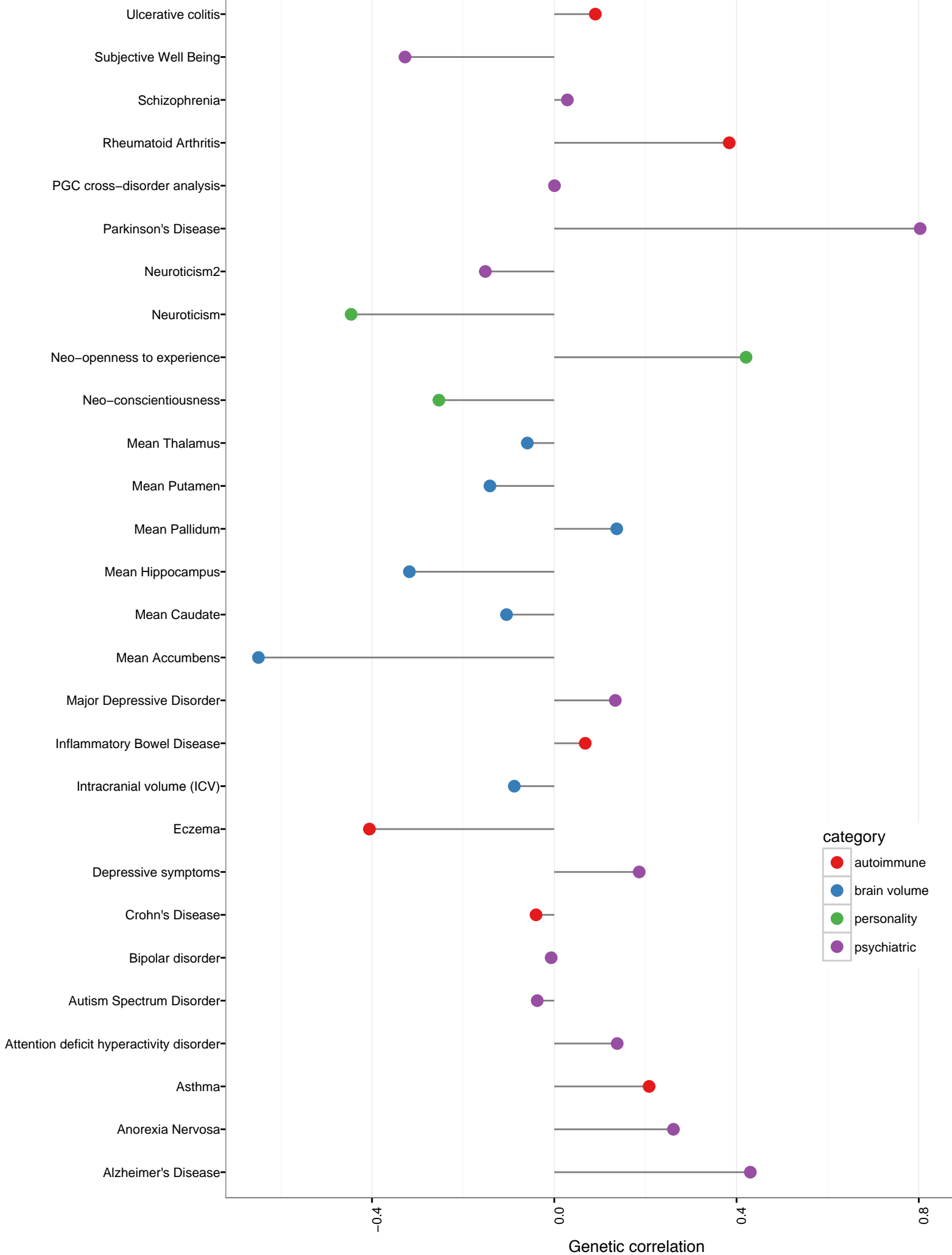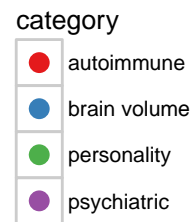

Supplement: Supplementary file 1 — Figure S1 [file 41398_2018_198_MOESM1_ESM.pdf]

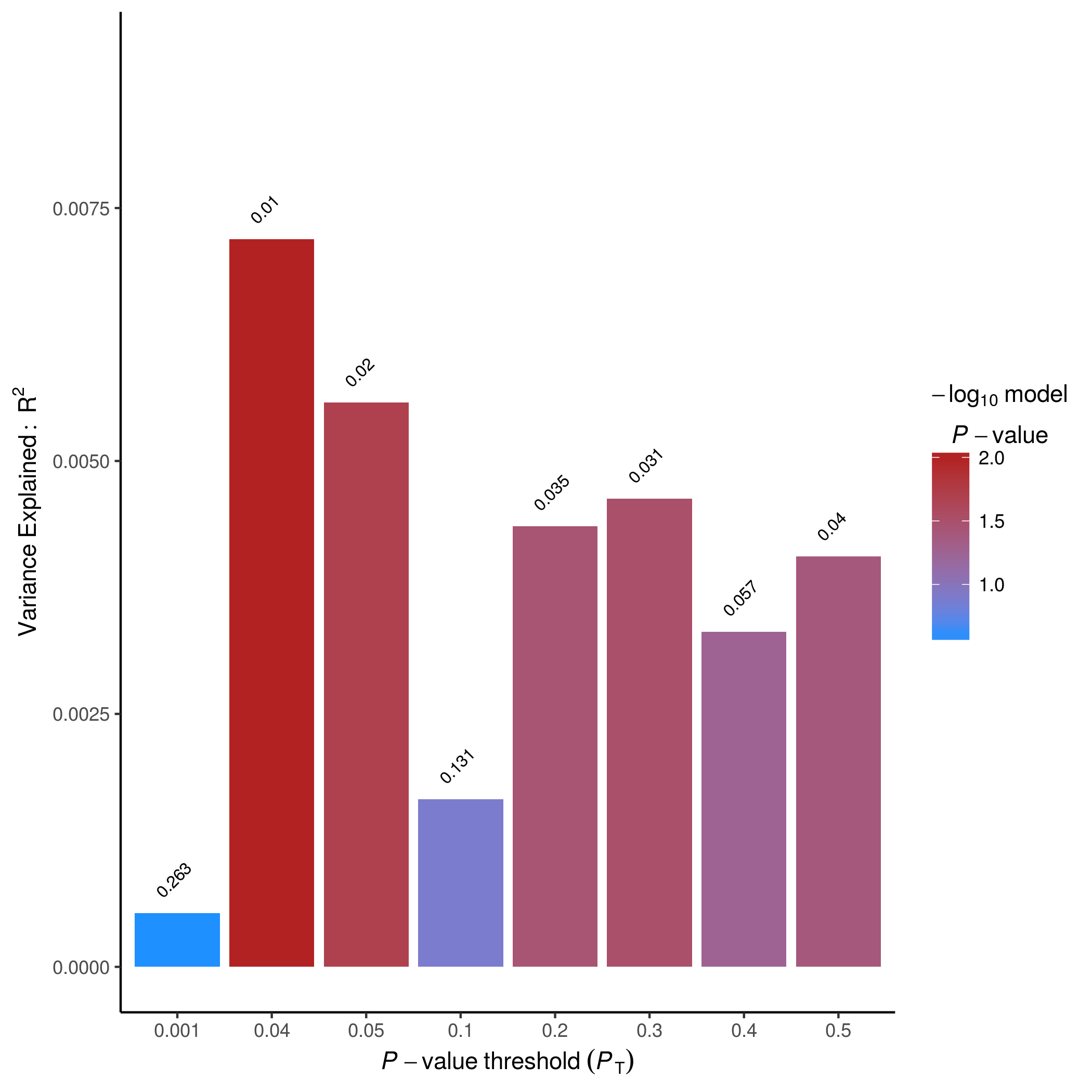

Supplement: Supplementary file 2 — Figure S2 [file 41398_2018_198_MOESM2_ESM.png]

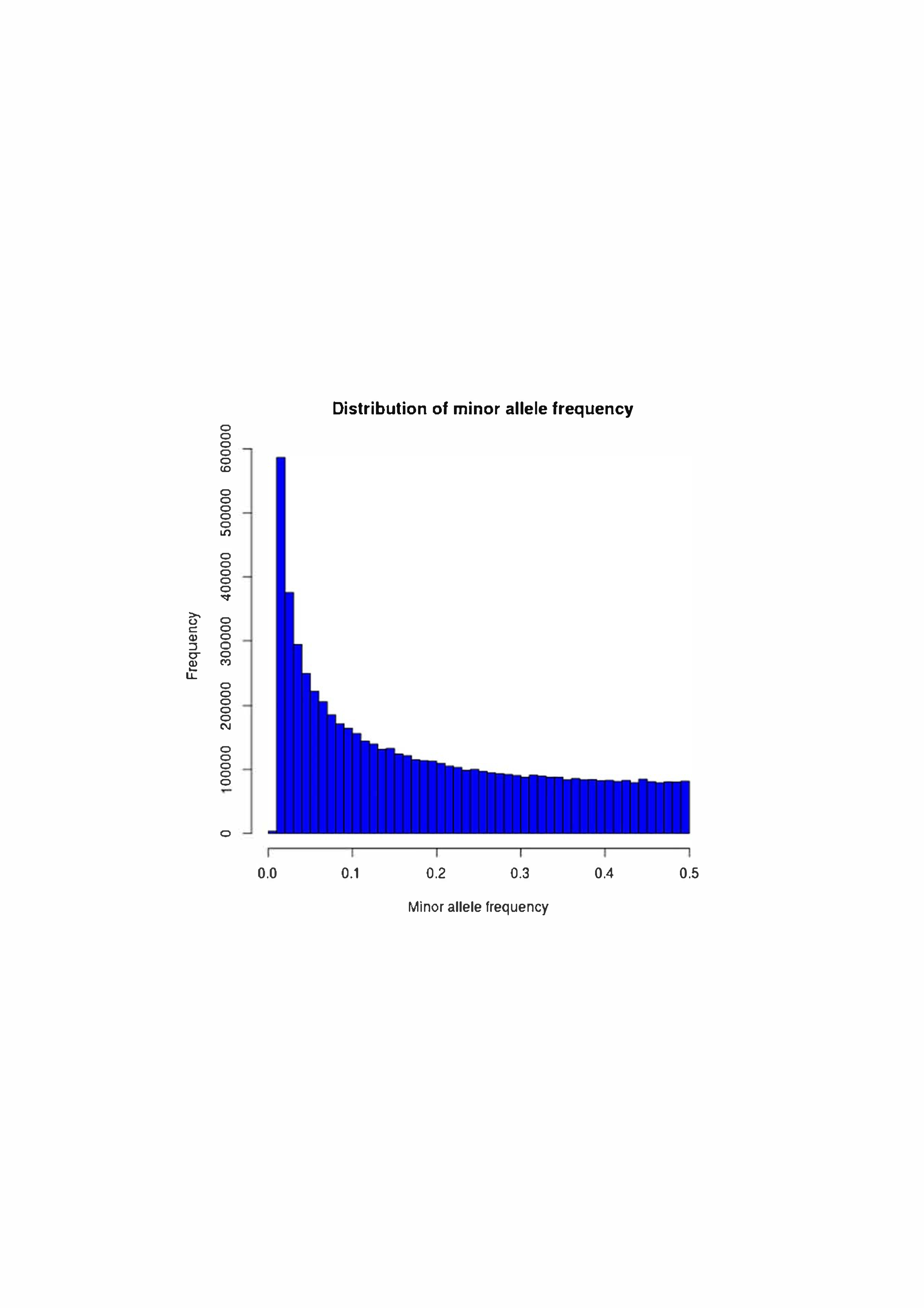

Supplement: Supplementary file 3 — Figure S3 [file 41398_2018_198_MOESM3_ESM.png]
